# Electrocardiographic ST-segment elevation with prominent R waves in precordial leads

**DOI:** 10.1007/s12471-022-01696-6

**Published:** 2022-05-10

**Authors:** A. Y. Andreou, A. R. Pérez-Riera

**Affiliations:** 1 Department of Cardiology, Limassol General Hospital, Limassol, Cyprus; 2 University of Nicosia Medical School, Nicosia, Cyprus; 3 Centro Universitario Saúde ABC, Santo André, SP Brazil; 4 Uninove University campus Mauá, São Paulo, Brazil

## Answer

**Fig. 1:**
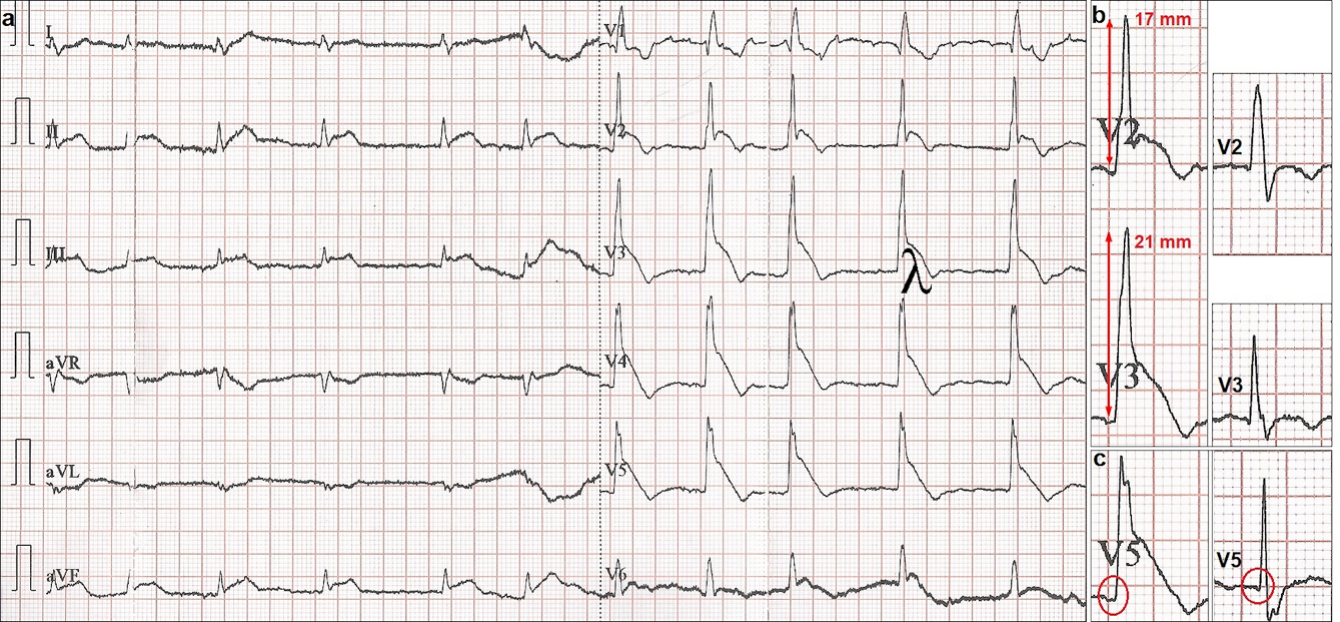
Electrocardiogram (ECG) during angina. **a** Atrial fibrillation, heart rate 74 bpm, QRS duration ~ 120 ms (no discernible change in relation to admission ECG), normal frontal QRS axis (75°), right bundle branch block and left septal fascicular block resulting in late and relatively unopposed predominantly left-to-right and posterior-to-anterior septal activation, with resultant prominent and anteriorly shifted mid-to-late QRS forces and leftward directed initial QRS forces, crescendo and decrescendo of R wave voltage in V1–V3 and V5–V6, respectively, and lambda-like ST-segment elevation pattern. **b** (*left panel*) R wave amplitude in V2–V3 > 15 mm. **c** (*left panel*) Absent septal Q waves in V5–V6. **b**, **c** (*right panels*) Admission ECG leads displaying R waves with markedly lower amplitude and septal Q wave in V5, respectively

**Fig. 2:**
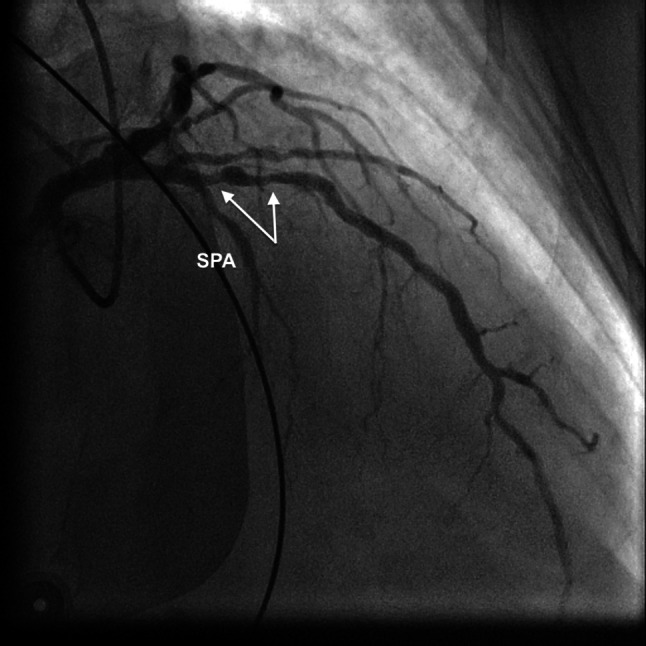
Conventional coronary angiography image depicting obstructive stenosis (*arrows*) in proximal left anterior descending coronary artery. First septal perforator artery (SPA), which arises immediately proximal to the stenosis, is also shown

Electrocardiography (ECG) during angina showed an ST-segment elevation resembling the Greek letter λ (lambda) and a striking increase in R wave amplitude (> 15 mm) in leads V2–V5 (prominent anterior QRS forces), loss of septal Q waves in V5–V6 and ST-segment elevation in II, aVF and III (Fig. 1). Emergency angiography revealed an obstructive stenosis in the proximal left anterior descending (LAD) coronary artery (Fig. 2), which was successfully treated with stenting.

ECG showed intermittent changes compatible with a conduction disturbance or block in the left septal fascicle (LSF), which is a third distinct division of the left bundle branch proceeding to the middle third of the left septal surface towards the apex [1]. Indeed, of all causes of prominent anterior QRS forces, only left septal fascicular block (LSFB) can manifest as intermittent ECG changes [1]. Durrer et al. demonstrated breakthrough activation through the LSF by using isolated, perfused human hearts, thereby for the first time proving its existence [2]. Ischaemia-induced LSFB has been associated with lesions in the proximal LAD coronary artery, the septal branches of which provide the entire blood supply of the LSF [1]. In our patient, ischaemic ST-segment elevation and LSFB were probably due to vasospasm at the site of the LAD artery lesion compromising blood flow in the first septal branch found in the immediate vicinity.

A precordial lambda-like ST-segment elevation comprises a prominent J wave merging with an elevated ST-segment and indicates an increased risk of malignant ventricular arrhythmias. ECG recognition of LSFB in acute coronary syndromes manifesting with this type of ST-segment elevation should alert physicians to the presence of a proximal LAD artery culprit lesion and prompt aggressive therapy including emergency angiography [3].
